# Pyroptosis in development, inflammation and disease

**DOI:** 10.3389/fimmu.2022.991044

**Published:** 2022-09-16

**Authors:** Yuhong Pan, Wenjun Cai, Juan Huang, Anchun Cheng, Mingshu Wang, Zhongqiong Yin, Renyong Jia

**Affiliations:** ^1^Research Center of Avian Disease, College of Veterinary Medicine, Sichuan Agricultural University, Chengdu, China; ^2^Institute of Preventive Veterinary Medicine, Sichuan Agricultural University, Chengdu, China; ^3^Key Laboratory of Animal Disease and Human Health of Sichuan Province, Chengdu, China

**Keywords:** pyroptosis, caspase-1, gasdermins, inflammation, disease

## Abstract

In the early 2000s, caspase-1, an important molecule that has been shown to be involved in the regulation of inflammation, cell survival and diseases, was given a new function: regulating a new mode of cell death that was later defined as pyroptosis. Since then, the inflammasome, the inflammatory caspases (caspase-4/5/11) and their substrate gasdermins (gasdermin A, B, C, D, E and DFNB59) has also been reported to be involved in the pyroptotic pathway, and this pathway is closely related to the development of various diseases. In addition, important apoptotic effectors caspase-3/8 and granzymes have also been reported to b involved in the induction of pyroptosis. In our article, we summarize findings that help define the roles of inflammasomes, inflammatory caspases, gasdermins, and other mediators of pyroptosis, and how they determine cell fate and regulate disease progression.

## Introduction

Cells can die through different pathways with unique morphological changes and physiological outcomes. Programmed cell death, such as apoptosis, necroptosis, and pyroptosis, are mediated by distinct sets of host proteins that coordinate various biological outcomes (1–3). Apoptosis is characterized by the activation of the caspase family, initiating caspases receiving intrinsic/extrinsic apoptosis signals and ultimately activating executioner caspases to trigger the cell death. During apoptosis, cell shrinks and divides into membrane-encapsulated apoptotic bodies that are normally phagocytosed by macrophages, resulting in non-inflammatory cell death.

Compared with apoptosis, pyroptosis is a necroptotic and inflammatory programmed cell death mode mediated by inflammatory caspases (3). Yet another type of necroptotic and inflammatory programmed cell death that executes independently of caspases is called necroptosis (4). Pyroptosis is a recently discovered cell death pathway that is regulated by various microbial infections (eg, *Legionella*, *Salmonella* and *Francisella*) *(* 5). Surprisingly, caspase-1, known to play a role in inflammation and cell survival, was identified as an important player in this novel cell death pathway. Caspase-1 was first known as IL-1β-converting enzyme (ICE), because of its ability to process inactive precursors of interleukin 1β (IL-1β) and IL-18 into mature and active cytokines (6). Furthermore, activation of caspase-1 not only produces inflammatory cytokines, but also leads to cell death characterized by rupture of the plasma membrane and release of pro-inflammatory cellular contents (7, 8). The term pyroptosis, derived from the Greek roots *pyro* (fire/fever) and *ptosis* (to-sis, falling), was first proposed by D’Souza et al. in 2001 to define this inflammatory cell death (9).

**Figure 1:**
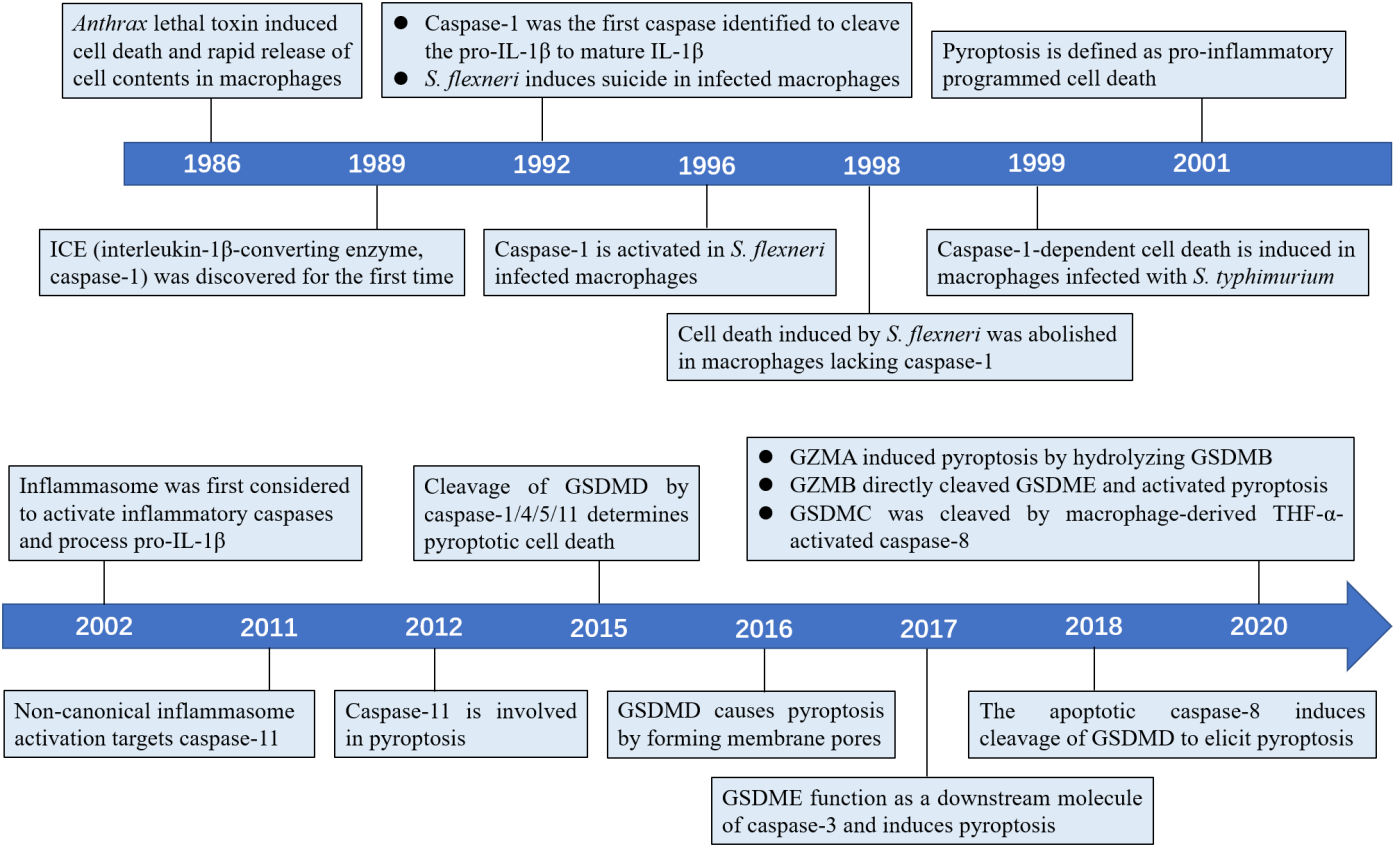
Timeline of the key discoveries about the pyroptosis.

In this timeline article, we discuss the seminal findings revealing the mechanistic of this newly discovered cell death pathway, including the molecules involved, such as inflammasomes, inflammatory caspases and gasdermins (**Figure 1**), and how inflammatory caspases determine cell fate in the context of microbial infection.

## Mechanism and features of pyroptosis

The earliest study of pyroptosis dates back to 1986, when Friedlander found that treatment of mouse macrophages with *Bacillus anthrax* lethal toxin (LeTx) triggers cell death and release of cellular contents (10). Caspase-1, an inflammatory caspase responsible for processing pro-IL-1β to mature IL-1β, was first discovered in 1989 by Cerretti et al. and Thornberry et al. (11–13). In 1992, Zychlinsky et al. observed suicide in macrophages infected with Gram-negative bacterial pathogen *Shigella flexneri*, the first time discovery of pyroptosis (14). In 1996, Chen et al. found that the invasion plasmid antigen B (ipaB) of *S. flexneri* can directly bind to caspase-1 and cause caspase-1 activation in macrophages (15). A new research in 1998 found *S. flexneri* inability to induce cell death in macrophages lacking caspase-1 (16). In addition, a study in 1999 found that macrophages infected with *Salmonella typhimurium* also induced caspase-1-dependent cell death (17). As previous studies have shown that caspase-1 can cleave the pro-inflammatory cytokines pro-IL-1β and pro-IL-18 (12, 18), the term pyroptosis was therefore coined in 2001 to indicate inflammatory caspase-1-dependent cell death (19). In 2002, the inflammasome was first identified to activate inflammatory caspases and process pro-IL-1β (20). Later, Petr et al. reported that non-canonical caspase-11 induces cell death in a caspase-1-independent manner following host infection with *Salmonella* (21). Today, the concept of pyroptosis has been expanded to include cell death performed by most inflammatory caspases, such as human caspase-4/5, and mouse *caspase-11* (2). Further, several studies have revealed that gasdermins serve as specific substrates of caspase-1/4/5/11 and as an effector molecule for pyroptosis (22, 23). In 2017, new research showed that GSDME can convert caspase-3-mediated apoptosis induced by TNF or chemotherapy drugs to pyroptosis, providing new insights into cancer chemotherapy (24, 25).

### Pyroptosis is a caspase-1-dependent cell death mode (Canonical pathway)

Pyroptosis, or caspase-1-dependent cell death is mediated by inflammasome assembly with cleavage of GSDMD and release of IL-1β/18 (26–28). Caspase-1 is initiated by multiple canonical inflammasome ligands and triggers pyroptosis (29). Inflammasomes are multi-protein signaling platforms that regulate inflammatory responses and coordinate host antimicrobial defense (30). In addition, inflammasomes have also been implicated in non-microbial diseases. Substantial evidence suggests that the inflammasome and its associated cytokines perform critical roles in tumorigenesis such as proliferation, invasion, and metastasis (31–33). Inflammasome assembly starts with cytoplasmic pattern recognition receptors (PRRs) that recognize pathogen-associated molecular patterns (PAMPs) and danger-associated molecular patterns (DAMPs) (34). Most inflammasomes are composed of three components: (i) the nucleotide-binding oligomerization domain (NOD)-like receptors (NLRs), (ii) an adapter apoptosis-associated speck-like protein (ASC), (iii) pro-caspase-1. All NLRs have a central NOD domain, most members have a C-terminal leucine-rich repeats (LRRs) domain and a variable N-terminal protein-protein interaction domain. Depending on whether N-terminal has a pyrin domain (PYD) or a caspase activation and recruitment domain (CARD), NLRs can be subdivided into NLRPs or NLRCs (35, 36). Certain members of the NLR family, including NLRP1, NLRP3, NLRC4, already possess the ability to form inflammasomes, while others, like NLRP2, NLRP6, NLRP12, are considered putative inflammasome sensors (37). The adaptor protein ASC contains two death-fold domains: an N-terminal PYD and a C-terminal CARD. ASC uses its PYD and CARD to bind to the PYD of NLR and CARD of pro-caspase-1, respectively, bridging upstream inflammasome sensor molecules to pro-caspase-1 (38). Notably, some PRRs directly recruit pro-caspase-1 due to the inclusion of CARD (30). After the inflammasome is successfully assembled, caspase-1 is hydrolyzed into two fragments, which then dimerize into active cleaved caspase-1 (39). On one side, caspase-1 cleaves pro-IL-1β/18 to generate mature IL-1β/18 (40). On the other side, caspase-1 specifically cleaves the linker between N-terminal gasdermin D (GSDMD-N) and C-terminal GSDMD (GSDMD-C), which is necessary for pyroptosis (23). Finally, GSDMD-N oligomerizes to form transmembrane and non-selective pores of ~10-14 nm in diameters, releasing cellular contents and inflammatory cytokines IL-1β and IL-18 and leading to pyroptosis (41, 42).

**Figure 2:**
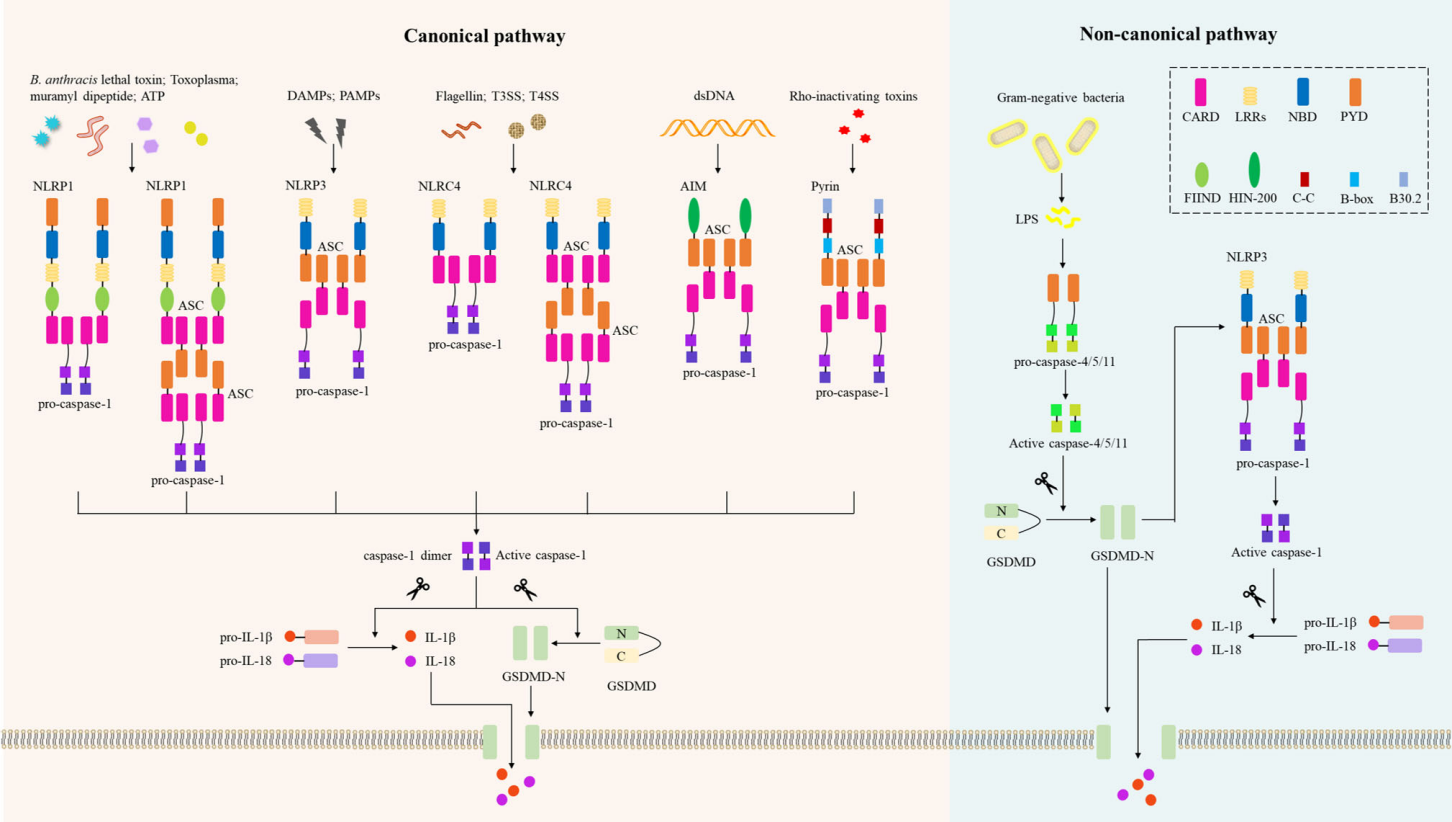
Inflammasomes in the canonical and non-canonical pyroptotic pathway. In the canonical pathway, danger-associated molecular patterns (DAMPs) or pathogen-associated molecular patterns (PAMPs) activate their respective inflammasome sensors, including NLRP1, NLRP3, NLRC4, AIM2, or Pyrin. *B. Anthrax* toxins, Toxoplasma, ATP, etc. activate the NLRP1 inflammasome, and NLRP1 recruits pro-caspase-1 *via* ASC or *via* CARD-CARD interaction. Multiple DAMPs and PAMPs activate the NLRP3 inflammasome, which subsequently recruits ASCs and pro-caspase-1. NLRC4 senses bacterial flagellin and T3SS/T4SS components and activates caspase-1 in an ASC-dependent or ASC-independent manner. The AIM2 inflammasome assembles when AIM2 senses host- or pathogen-derived dsDNA; the pyrin inflammasome is activated by Rho-inactivating toxins, and both AIM2 and pyrin activate caspase-1 in an ASC-dependent manner. Activated caspase-1 induces proteolytic cleavage of GSDMD to generate N-GSDMD, which then forms the GSDMD-N pore and induces pyroptosis. Activated caspase-1 also induces proteolytic maturation of IL-1β and IL-18 to their active forms, which are secreted through the GSDMD-N pore. In the non-canonical pathway, LPS from Gram-negative bacteria are recognized by caspase-4 or caspase-5 in human cells or caspase-11 in mouse cells. These activated inflammatory caspases directly cleave GSDMD and form GSDMD-N pores to trigger pyroptosis. The GSDMD-N-terminal fragment also activates the NLRP3 inflammasome and induces caspase-1-dependent maturation of IL-1β and IL-18. CARD, caspase recruitment domain; LRRs, leucine-rich repeats; BD, nucleotide-binding and oligomerization domain; PYD, pyrin domain; N FIIND, a functional-to-find domain; HIN-200, hematopoietic interferon-inducible nuclear protein 200; CC, coiled-coil; B-box, B-box-type zinc finger; AIM2, absent in melanoma 2; ASC, apoptosis-associated speck-like protein containing a caspase recruitment domain; GSDMD, gasdermin D; LPS, lipopolysaccharide.

To date, several receptor proteins of the NLRs have been shown to assemble the inflammasome, including NLRP1, NLRP3, NLRC4 (NLRP2/6/12), and two cytoplasmic PRRs: absent in melanoma 2 (AIM2) and pyrin (30) (**Figure 2**). Compared to NLRP3, NLRP1 has additional function-to-find domain (FIIND) and CARD domains at C-terminal (43). Thus, NLRP1 does not require ASC to activate pro-caspase-1, resulting in IL-β secretion (44). Human NLRP1 has three paralogs in mice: *NLRP1a/b/c*, but these *NLRP1s* in mice lack PYD. Using an *in vitro* system consisting of recombinant proteins, it was confirmed that NLRP1 in humans can be activated by ATP and muramyl dipeptide (45). Additionally, inhibitors of dipeptidyl peptidases 8 and 9 (DPP8/9) initiate the NLRP1 inflammasome, triggering pyroptosis of resting lymphocytes in humans and rodents (46). Importantly, NLRP1 acts as an innate immune sensor to produce IL-18 in response to metabolic stress, thereby preventing obesity and metabolic syndrome (47), and *NLRP1b* is also an innate immune sensor of Toxoplasma infection (48). Notably, *NLRP1b* activates caspase-1 to induce macrophage pyroptosis in response to *B. anthracis* lethal toxin (LeTx) (49), and the protease component of LeTx is required for inflammasome activation, whereas anthrax lethal factor (LF) was reported to cleave *NLRP1b* (50).

Several molecular and cellular events have been proposed as triggers for NLRP3 inflammasome, such as K^+^ efflux, Ca^2+^ signaling, the production of reactive oxygen species (ROS), mitochondrial dysfunction, and lysosomal disruption (31). NIMA-related kinase 7 (NEK7) is an essential protein that mediates NLRP3 inflammasome assembly and activation downstream of K^+^ efflux (51), but prevents inflammasome formation during the mitotic phase of the cell cycle (52). New data demonstrate that NEK7 mediates NLRP3 inflammasome activation by bridging adjacent NLRP3 subunits (53). Notably, different NLRP3 stimuli all cause disassembly of trans-Golgi network (TGN), and NLRP3 is recruited to dispersed TGN (dTGN) *via* phosphatidylinositol 4-phosphate (PtdIns4P) on dTGN to activate downstream signaling cascades (54). Importantly, Szekanecz et al. (55) found that NLRP3 inflammasome-IL-1 pathway could serve as therapeutic targets for gout; another study found that cardamonin from a herbal medicinal prevents LPS-induced septic shock by inhibiting the NLRP3 inflammasome (56). Therefore, NLRP3 is emerging as a potential drug target for the treatment of inflammatory diseases (57).

NAIPs (NLR family apoptosis inhibitory proteins) were identified as immunosensors required for NLRC4 inflammasome activation (58). Human NAIP2 and NAIP5 recognize bacterial flagellin and type III/IV secretion system (T3SS/T4SS) apparatus components, respectively, to activate NLRC4 inflammasome-mediated innate immunity (59), resulting in cleavage of pro-caspase-1, and GSDMD-N pores formation and pyroptosis (60). Compared with human NAIPs, mouse *NAIP1*, *NAIP2*, *NAIP5* and *NAIP6* recognize the needle of T3SS, the inner rod of the T3SS, flagellin and flagellin, respectively (61). Of note, several gain-of-function mutations in NLRC4 have been implicated in the development of autoinflammatory disease (62).

AIM2 has been identified as a DNA receptor that induces inflammasome formation (63). AIM2 contains an N-terminal PYD and a C-terminal DNA-binding HIN-200 domain, senses cytoplasmic negatively charged double-strand DNA (dsDNA) through its positively charged HIN-200 domain, and interacts with ASC through its PYD (64). The ASC speck recruits pro-caspase-1 *via* CARD-CARD interaction, driving pro-IL-1β/18 proteolytic cleavage and pyroptosis (65). The release of microbial DNA into the cytoplasm leads to activation of the AIM2 inflammasome during infection by *Listeria*, *Francisella*, *Mycobacterium*, vaccinia virus, mouse cytomegalovirus, Plasmodium and Aspergillus (66). Similar to other inflammasomes, AIM2 inflammasome is precisely regulated in cells and has been reported to be negatively regulated by interferon-inducible protein p202 in mBMDMs (67). Notably, IFN-I boosts AIM2-dependent caspase-1 activation by promoting AIM2 protein expression (68). However, improper recognition of self-DNA by AIM2 can lead to the development of autoimmune and inflammatory diseases such as psoriasis, arthritis and dermatitis (65).

Pyrin is a candidate PRR that binds to ASC adaptor to form a caspase-1-activating complex by sensing pathogen modifications and inactivation of Rho GTPase (69). Interestingly, pyrin does not directly recognize molecular patterns (PAMPs and DAMPs), but responds to infection-induced disturbances in cytoplasmic homeostasis, these perturbations are called ‘homeostasis-altering molecular processes’ (HAMPs) (70). Rho-inactivating toxins including *Vibrio parahaemolyticus* VopS, *Clostridium difficile* glycosyltransferase TcdB, and *Clostridium botulinum* ADP-ribosylating C3 toxin could trigger the assemble of pyrin inflammasome. Inactivation of RhoA by *Yersinia* effectors YopE and YopT (*Yersinia* ectoprotein) triggers the pyrin inflammasome (71), while YopM was identified as a microbial inhibitor of the pyrin inflammasome (72). Furthermore, mutations in B30.2/SPRY domain of pyrin lead to pathogen-independent activation of pyrin and trigger autoinflammatory disease familial Mediterranean fever (FMF). Another dominantly inherited autoinflammatory disorder distinct from FMF, characterized by recurrent childhood-onset neutrophilic dermatosis, is induced by mutations in the gene encoding pyrin, MEFV (S242R) (73).

The astrocytic NLRP2 inflammasome is a vital component in CNS inflammatory responses, suggesting NLRP2 inflammasome could become a therapeutic target for suppressing CNS injury-induced inflammation (74). Growing evidence suggests that microbial signals (eg. type I IFNs), metabolic signals (eg. PPARγ activators), and microbial components (metabolites, RNA, lipoteichoic acid (LTA), LPS) can activate the NLRP6 inflammasome (75). Importantly, microbiota-related metabolites taurine, histamine and spermine shape the host-microbiota interface by co-regulating NLRP6 inflammasome signaling and epithelial IL-18 secretion (76). NLRP12 induces the production of inflammasome-dependent cytokines IL-1β/18 following *Yersinia* or *Plasmodium salina* infection, thereby preventing severe infections by these pathogens. Of note, NLRP12 is also a core component in maintaining intestinal inflammation and intestinal homeostasis. In contrast, NLRP12 exhibits as a negative mediator of NF-κB and MAPK signaling during infection with *S. typhimurium*, *Klebsiella pneumoniae*, *Mycobacterium tuberculosis*, or vesicular stomatitis virus, as well as during colon tumorigenesis. NLRP12 also negatively regulates canonical and non-canonical pathways in T cells and contributes to the exacerbation of autoimmune diseases. We speculate that the functional shift of NLRP12 as an inflammasome or negative regulator depends on the environment (77).

Interestingly, caspase-1-driven, pore-induced pyroptosis induces multifaceted defense against intracellular bacteria that is facilitated by pathogens entrapment in cellular debris, termed pore-induced intracellular traps (PIT) (78). Another study found that during inflammasomes activation, cells can undergo pyroptosis and release cytokines without triggering cell death, and Toll-like receptor adapter protein SARM may be participated (79). It is worth noting that thymocytes and macrophages from caspase-1-deficient animals typically undergo apoptosis (40). Furthermore, *Francisella tularensis* activates AIM2/ASC-dependent caspase-3-mediated apoptosis in macrophages lacking caspase-1 (80). Analysis of macrophages and primary cells treated with LPS plus nigericin or *S.typhimurium* indicated apoptosis became evident in GSDMD^(-/-)^ cells, showing a inhibition of apoptosis by pyroptosis, and caspase-1 may devote to the apoptotic caspases activation in GSDMD^(-/-)^ cells (81). These findings highlight the crosstalk between pyroptosis and apoptosis pathways and underscore the versatility of the inflammasome.

### Caspase-1-independent pyroptosis (Non-canonical pathway)

In the non-canonical pyroptotic pathway, human caspase-4/5 and the mouse homolog caspase-11 bind directly to lipopolysaccharide (LPS) of Gram-negative bacteria with high specificity and affinity (82). Binding to LPS is mediated by the CARD of the caspase, resulting in autoproteolysis and oligomerization of caspase (83). Subsequently, GSDMD is cleaved by inflammatory caspase-4/5/11 to generate N-terminal fragments, and GSDMD-N forms pores that impair cell membrane integrity (84), leading to K^+^ efflux (23), induction of NLRP3 inflammasome assembly, and ultimately pyroptosis (**Figure 2**) (85, 86). Although caspase-4/5/11 can’t cleave pro-IL-1β/18, they could regulate IL-1β/18 maturation and secretion by activating NLRP3/caspase-1 signaling (22, 85, 87). During non-canonical inflammasome signaling, caspase-11 generates active caspase-11 protease by self-cleaving at D285, which mediates GSDMD cleavage and NLRP3-dependent IL-1β production (88). Importantly, caspase-11 dimerization is required to initiate its auto-cleavage ability. Like *caspase-1*-deficient mice (89), *caspase-11* mutant mice protect against LPS-induced endotoxic shock (40), besides, IL-1α and IL-1β production following LPS stimulation is blocked in *caspase-11* mutant mice. These results indicate that caspase-11 is necessary for the activation of caspase-1 (90). Additionally, mice lacking *caspase-11* had reduced numbers of apoptotic cells and lacked *caspase-3* activation, suggesting that caspase-11 is also responsible for activating caspase-3 under certain pathological conditions (91).

In the face of cytoplasmic invading bacteria, caspase-4 activation *via* guanylate-binding proteins (GBPs) platform is critical for inducing GSDMD-dependent pyroptosis and processing of IL-18 (92). Yang et. al (93) reported that cytosolic LPS triggers caspase-11-dependent pannexin-1 channel cleavage, resulting in ATP release and subsequent activation of purinergic P2X7 receptors to mediate cytotoxicity. Cytosolic LPS-induced pyroptosis was abolished in the absence of P2X7 or pannexin-1. These data show the indispensable roles of pannexin-1 and P2X7 downstream of caspase-11 in pyroptosis. Of note, the oxidized phospholipid 1-palmitoyl-2-ara-chidonoyl-sn-glycero-3-phosphorylcholine (oxPAPC) competes with LPS to bind caspase-4 and caspase-11, thereby inhibiting LPS-induced macrophages pyroptosis, but not in dendritic cells (94). In addition, an epithelial cell-intrinsic non-canonical inflammasome has an important role in antimicrobial defense of intestinal mucosal surface, driving infected cells excretion to limit the intraepithelial proliferation of pathogens (95, 96). These studies suggest that non-canonical activation of inflammatory caspases by LPS initiates a novel paradigm for innate immunity.

## Gasdermin, the executioner of pyroptosis

### GSDMD as a common executor of pyroptosis

GSDMD is required for pyroptosis induction and IL-1β/18 secretion in both canonical and non-canonical inflammasome responses (81). GSDMD contains two domains, 31 kDa N-terminal (GSDMD-N) and 22 kDa C-terminal (GSDMD-C), which are linked with a long loop (23). Inflammasome-associated caspases (caspase-1/4/5/11) cleaves the linker between the GSDMD-N and GSDMD-C, which is necessary to induce pyroptosis, while its C-terminal domain binds the N-terminal domain to suppress pyroptosis (97). The cleavage releases intramolecular suppression of GSDMD-N, showing pyroptosis-inducing ability. Studies have also found that apoptotic caspase-8 is able to cleave GSDMD to trigger pyroptosis (98, 99). Since GSDMD-N exhibits strong and specific binding to phosphoinositide and cardiolipin, which are known to exist in mammalian cytoplasmic membranes and bacterial inner membranes, respectively, suggesting that GSDMD-N could directly target the membrane and lyse it (100). GSDMD-N migrates to plasma membrane to form functional pores that disrupt the integrity of the cell membrane and release cytokines including IL-1β and IL-18. The gasdermin pores have an diameter of 10~14 nm and contain 16 symmetrical protomers (101). GSDMD-N can also destroy bacteria, including *Escherichia coli*, *Bacillus megaterium protoplasts*, and *Staphylococcus aureus* (41). Additionally, the N-terminal of GSDMD also initiates activation of NLRP3-dependent inflammasome (22, 23, 102), most likely requiring K^+^ efflux induced by GSDMD-N-induced membrane pores (86). It is worth noting that GSDMD-deficient cells were resistant to pyroptosis induced by LPS and known canonical inflammasome ligands, with reduced IL-1β release despite intact caspase-1 processing (23). But GSDMD-deficient cells may eventually die, possibly because of caspase-3/7 cleavage by caspase-1 (103, 104). In addition to its central role in pyroptosis, GSDMD also plays an essential function in NETosis (105). Caspase activation and specific recognition are the complete molecular mechanisms of GSDMD-mediated pyroptosis. Different caspases have different selective preferences for peptide sequences and thus have different substrate recognition profiles. Unlike caspases in the apoptotic pathway, GSDMD is the only physiological substrate known to caspase-4/5/11. The specific self-cleavage of caspase-4 and caspase-11 occurs at different subunits, which is critical for their activation and GSDMD cleavage (106). GSDMD is cleaved at the Asp275 site, which can release self-inhibition and initiate pyroptosis. Interestingly, caspase-3/7 cleaved GSDMD at Asp87 site, causing inactivation of the pyroptotic activity of GSDMD (107, 108). Succination of GSDMD on Cys192 prevents its processing and oligomerization, which limits pore formation, cytokine release, and cell death (109).

### Other members of the gasdermin family

Other members of gasdermin (GSDM) family are becoming regulators of programmed cell death in various processes regulating cell proliferation and differentiation. The GSDM protein family is conservative in vertebrates and includes six homologs in human, termed GSDMA, GSDMB, GSDMC, GSDMD, GSDME/DFNA5, and DFNB59 (**Table 1**). Although mouse lacks GSDMB, it has three GSDMA homologs and four GSDMC homologs besides GSDMD. In addition to DFNB59, all GSDMs have two domains with a variable linker, and share similar auto-inhibitory structures. DFNB59 retains GSDM N-terminal sequence homology, while is biased at its C-terminal. Furthermore, GSDME and DFNB59 are expressed in heart, brain, kidney and inner ear; on the contrary, the remaining GSDMs are mainly expressed in the skin system and the gastrointestinal tract (epithelium) (110). The GSDM-N domains of GSDMA/*GSDMA3*, GSDMB, GSDMC, and GSDME, but not full-length proteins, all could trigger pyroptosis in mammalian cells and eliminate bacteria (23), the pore-forming activity of GSDM-N domains are necessary for pyroptosis (100).

**Table 1:** Properties and functions of gasdermin family proteins.

Human gene	Alises	Mouse gene	Domain^a^	Pore-forming activity	RNA expression pattern^b^	Activation mechanism	Disease
GSDMA	GSDM, GSDM1, FKSG9, gasdermin A	Gsdma1-3	N+C	Yes	skin of abdomen, placenta, gastric mucosa, smooth muscle tissue, appendix, gallbladder, duodenum, lymph node, rectum, urinary bladder	Unknown	Alopecia, asthma
GSDMB	GSDML, PRO2521, PP4052, gasdermin B, GSDMB-1	Absent	N+C	Yes	rectum, right lobe of liver, duodenum, cerebellar hemisphere, jejunal mucosa, body of stomach, right uterine tube, gallbladder, spleen, body of pancreas	GZMA	Asthma and other autoimmune diseases
GSDMC	MLZE, gasdermin C	Gsdmc1-4	N+C	Yes	zone of skin, skin of abdomen, spleen, vagina, skeletal muscle tissue, minor salivary gland, gastrocnemius muscle, left ventricle, thymus, urinary bladder	Caspase-8	Breast cancer
GSDMD	DF5L, DFNA5L, GSDMDC1, FKSG10, gasdermin D	Gsdmd	N+C	Yes	spleen, right lobe of liver, duodenum, blood, right lobe of thyroid gland, upper lobe of left lung, right lung, appendix, lymph node, canal of the cervix	Caspase-1/4/511	Sepsis
DFNA5	GSDME, ICERE-1, deafness associated tumor suppressor, gasdermin E	Dfna5	N+C	Yes	germinal epithelium, jejunal mucosa, stromal cell of endometrium, endothelial cell, optic nerve, placenta, oocyte, secondary oocyte, middle temporal gyrus, spinal cord	Caspase-3	Deafness
DFNB59	pejvakin, PJVK, deafness, autosomal recessive 59	Dfnb59	N	Unknown	sperm, anterior pituitary, right lobe of liver, brodmann area 9, left lobe of thyroid gland, gastric mucosa, right uterine tube, canal of the cervix, right lobe of thyroid gland, tibial nerve	Unknown	Deafness

^a^N and C stand for gasdermin-N and gasdermin-C, respectively.

^b^Expression data taken from [95-99, 104-122] as well as from Bgee (www.bgee.org.com).

Notably, the expression of GSDMA was decreased in gastric and esophageal cancers, suggesting its tumor suppressor effect (111). Further, gain-of-function mutations of *GSDMA3* that lead to skin defects and alopecia disrupt autoinhibition, resulting in *GSDMA3-N* domain-induced pyroptosis (23). *GSDMA3-N* also regulates mitochondrial oxidative stress by targeting mitochondrial (112). Human GSDMB polymorphisms are strongly associated with autoimmune diseases including inflammatory bowel disease (IBD) and asthma (113, 114). Among them, GSDMB cleavage by lymphocyte-derived granzyme A (GZMA) exerts its pore-forming ability and promotes pyroptosis, a killing mechanism that enhances antitumor immunity (115). Interestingly, GSDMB is regarded as an oncogene because it is overexpressed in several cancers including gastric cancer (116), breast cancer (117) and cervical cancer (118). Therefore, a more in-depth study on the role of GSDMB in pyroptosis and cancers are required. Although artificial mutated GADMC can cause cytotoxicity in cell lines, the understanding of GSDMC is limited (100). In addition, the expression of GSDMC is related to metastatic melanoma, whereas GSDMD expression is detected in gastric cancers (GCs) (111). More recently, PD-L1 converts TNF-mediated apoptosis into pyroptosis in breast cancer cells. Under the stimulation of TNF-α, caspase-8 specifically lyses GSDMC to produce N-GSDMC, and forms pores on the cell membrane to induce pyroptosis. In addition, antibiotic chemotherapy drugs can also trigger caspase-8/GSDMC-mediated pyroptotic death in breast cancer cells (119). GSDME, also called deafness autosomal dominant 5 (DFNA5), genetic mutations in GSDME induce nonsyndromic hearing loss in humans (120, 121). In line with gain-of-function of the deafness mutation, GSDME knockout mice show no hearing impairment (122). Of note, GSDME was identified as a key molecule regulating apoptotic cell disassembly and progression to pyroptosis (24). GSDME-N permeabilizes mitochondrial membranes, releases cytochrome c (Cyt C) and activates apoptosomes. Like GSDME-N, GSDMD-N also permeates mitochondria, linking activation of apoptotic bodies to downstream inflammasome activation (123). Importantly, GSDME is emerging as an important tumor suppressor gene and a valuable molecular marker of human cancers, since GSDME regulated pyroptosis conduces to the cytotoxicity of various chemotherapeutic agents (124, 125). Among them, GSDME is specifically cleaved by caspase-3 in its linker, resulting in a GSDME-N fragment that penetrates the membrane and induces pyroptosis, providing new insights into cancer chemotherapy (25). Additionally, the cell-killer granzyme B (GZMB) is released to tumor cells by cytotoxic lymphocytes, followed by cleavage of GSDME by GZMB, inducing tumor cell pyroptosis (126). DFNB59 is also associated with deafness and is first human gene involved in nonsyndromic deafness caused by neuronal defects (120). It is worth noting that DFNB59 is also peroxisomes-associated and participated in its oxidative stress-induced proliferation (127).

## Caspases and disease

### Caspase-1 in host response and disease pathology

Caspase-1-meidated innate immunity plays a role in the control of various bacterial infections, including *S. typhimurium* (128), *S. flexneri* (129), *Listeria* (130), *Legionella pneumophila* (131, 132), etc (**Table 2**). Caspase-1 substrates IL-1β and IL-18 are associated with restriction of *S. typhimurium* invasion, with IL-18 being important for resistance to systemic infection, while IL-1β is involved in the intestinal stage of the disease. Thus, caspase-1 is essential for the host’s innate immune defense *S. typhimurium* (146). Furthermore, *casp1*^(-/-)^ mice were more susceptible to *S. typhimurium* invasion compared to *casp1^(-/-)^ casp11*^(-/-)^ mice, while both strains had elevated bacterial loads compared to wild-type mice, while bacterial quantities were similar between wild-type and *casp11*^(-/-)^ mice, suggesting that caspsse-1, but not caspase-11, is protective against *S. typhimurium*, and caspase-11-dependent cell death is detrimental to host in the absence of caspase-1-mediated innate immunity (21). Although *S. typhimurium* normally resides in the vacuole, a specific mutant (sifA) enters the cytoplasm. The mutants trigger caspase-11 with elevated clearance of *S. typhimurium* sifA, suggesting caspase-11 is essential for innate immunity against cytosolic but not vacuolar bacteria (135). Another study reported caspase-11 is needed for intestinal tissue secretion of IL-18 in response to *S. typhimurium* infection and to control cecal bacterial populations (95). Importantly, *S. typhimurium* lacking the flagellin genes *fliC* and *fljB* exhibit a weakened ability to cause inflammation *in vivo*, probably due to its lack of motility and/or impaired activation of flagellin-sensing NLRC4-caspases-1 inflammasome and TLR5 (147–150). In contrast, *S. typhimurium* strains that overexpress flagellin because of lack of YdiV, a transcriptional suppressor of flagellin-encoding gene *fliC*, cause macrophage pyroptosis and induce elevated levels of IL-1β and TNF, ultimately fail in mouse tissue colonization (133).

**Table 2:** Inflammatory caspases are involved in defense against multiple pathogen infections.

Pathogens	Microbial species^a^	Caspases	Notable cytokines	*In vivo* or *in vitro*	Cell lines	References
*Salmonella typhimurium*	G-	caspase-1	IL-18, IL-1β	*in vivo*		(128)
		caspase-1	IL-1β, TNF-α	*in vivo*		(133)
		caspase-1	ROS	*in vivo*		(134)
		caspase-11	IL-18	*in vivo*		(95)
		caspase-4	IL-18	*in vivo*		(95)
*Salmonella typhimurium sifA*	G-	caspase-11	/	*in vivo*		(135)
*Burkholderia thailandensis*	G-	caspase-1	IL-18, IFN-γ	*in vivo*		(136)
		caspase-1	ROS	*in vivo*		(134)
		caspase-11	/	*in vivo*		(135, 136)
		caspase-1/11	IL-1β	*in vitro*	BMMs	(135)
		caspase-4	/	*in vivo*		(136)
*Francisella tularensis*	G-	caspase-1	IL-18, IL-1β	*in vivo*		(137)
		caspase-1	IL-18, IL-1β	*in vitro*	macrophage	(138)
		caspase-1	IL-1β	*in vitro*	monocytes	(139)
		caspase-1	IL-1β	*in vitro*	dendritic cells	(140, 141)
*Listeria monocytogenes*	G+	caspase-1	IL-18, IFN-γ	*in vivo*		(130)
		caspase-1	IL-18	*in vivo*		(142)
		caspase-1	IL-18, IL-1β	*in vitro*	macrophage	(143)
*Legionella pneumophila*	G-	caspase-1	ROS	*in vivo*		(134)
		caspase-1	IL-18, IL-1β	*in vitro*	macrophage	(131)
*Burkholderia pseudomallei*	G-	caspase-11	/	*in vivo*		(135)
		caspase-1/11	IL-1β	*in vitro*	BMMs	(135)
*Anaplasma phagocytophilum*	G-	caspase-1	IL-18, IFN-γ	*in vivo*		(144)
*Escherichia coli*	G-	caspase-4	IL-18	*in vivo*		(95)
*Candida albicans*	Fungus	caspase-1	IL-18	*in vivo*		(145)

^a^G+ stands for Gram-positive bacteria and G- for Gram-negative bacteria.

Caspase-1 is also involved in the cytoplasmic recognition of *L. pneumophila* flagellin by mouse macrophages, thereby limiting their infection (131). The NLRC4-caspase-1 inflammasome can trigger an IL-18-driven natural killer (NK) cell response that directs sterilizing immunity to intracellular bacterial pathogens such as *Listeria* (142, 143). Furthermore, activation of IL-18 by caspase-1 promotes IFN-γ to initiate caspase-11 and clearance of the cytoplasmic invasive bacterium *Burkholderia thailandensis* (136), whereas innate immunity to *F. tularensis* depends on the ASC/caspase-1 axis (137). New research finds that mice lacking *caspase-1* are more susceptible to *Francisella* compared to mice lacking *IL-1β* and *IL-18*, suggesting cell death itself and other *caspase-1*-dependent pathways help restrict infection (151). Additionally, caspase-1 and ASC are indispensable for IFN-γ to control *Anaplasma phagocytophilum* infection (144). Monosodium urate (MSU) or calcium pyrophosphate dihydrate (CPPD) crystals are associated with inflammatory responses in gout and pseudogout. MSU and CPPD bind to the NLRP3 inflammasome that activate caspase-1, producing active IL-1β/18 (152). Other findings suggest that pyroptosis protects against bacterial infections even in the absence of IL-1α/β and IL-18 (96). For example, caspase-1-mediated pyroptosis releases bacteria in macrophages, such as *B. thailandensis*, *L. pneumophila*, and *S. typhimurium*, which are taken up by neutrophils and absorbed by intracellular ROS, independent of IL-1β/18 (134). Another mechanism by which pyroptosis clears bacteria is to crowd out infected cells from tissues. Enterocytes infected with *S. typhimurium* induce caspase-1/11 inflammasome activation, leading to the physical extrusion of infected enterocytes from intestine (95, 96).

Activation of caspase-1 also regulates the progression of adaptive immune response, and IL-18 can promote the differentiation of CD4^+^ T cells and the production of IFN-γ (153). Among them, caspase-1-dependent IL-18 production was used to express sustained Th1 responsiveness to *Candida albicans* (145). Whereas, lack of caspase-1 down-regulates CD4^+^ T cell-induced IFN-γ after stimulation with *A. phagocytophilum in vitro* (144). In contrast, *Listeria*-specific acquired immunity was normal in *casp1*^(-/-)^ mice, while the cytokine profile is skewed towards T(h)2 compared to wild-type mice (130). In addition, aluminum-containing adjuvants can stimulate caspase-1 to activate a robust humoral immune response (154), and can also induce the release of IL-1β/18 of macrophages and dendritic cells to establish adaptive cellular immunity (154, 155), among which, the NLRP3 inflammasome is indispensable for alum-induced pro-caspase-1 processing (156, 157). Nonetheless, the regulation of antibody production by caspase-1 remains controversial (156, 157).

While pyroptosis protects the host against infections, inappropriate or excessive caspase-1 activity and pyroptosis may be detrimental. Unexamined inflammasome activation inhibits both IL-17-mediated antimicrobial responses against *Pseudomonas aeruginosa* and CD8^+^ T cell-mediated immune responses against *Listeria* (158–160). Additionally, overactivation of caspase-1 has been associated with myocardial infarction (5), ischemic brain injury (161), chronic colitis (162), and acute renal failure (163). It is also involved in triggering endotoxic shock (164) and exacerbating neurodegenerative diseases such as Huntington’s disease (165). Since caspase-1 deficiency or pharmacological inhibition prevents inflammation, cell death, and organ dysfunction related to these diseases, caspase-1 is a potential therapeutic target. Of note, neutralization of caspase-1-activated cytokines IL-1β/18 failed to achieve the protective effect of caspase-1 deficiency against sepsis and renal failure, suggesting that caspase-1 has other roles in disease besides activating cytokines effect (163, 166, 167).

### Caspase-4/5/11 in host response and disease pathology

In LPS-transfected human monocyte THP-1 cell line, caspase-4, but not caspase-5, has been found to promote IL-1β production and cell death. However, caspase-4 and caspase-5 exhibited non-redundant functions in THP-1 cell against *S. typhimurium* infection (85, 168). Likewise, *casp11*^(-/-)^ mice expression human caspase-4 were observed to support caspase-1 activation and IL-1β/18 secretion in face of endotoxin LPS in bone marrow-derived macrophages, whereas *casp11*^(-/-)^ mice are generally resistant to LPS-induced lethality. Unlike *casp11*^(-/-)^ mice, *casp11*^(-/-)^ mice expressing caspase-4 are protected from lethal *B. thailandensis* invasion (136). It is worth noting that the *enteropathogenic E. coli* (EPEC) virulence factor NleF binds caspase-4 and restricts the processing of caspase-4 and IL-18 in intestinal epithelial cells (IECs) (169). Further studies showed that caspase-4 is required for IL-1α secretion and pyroptosis in human macrophages infected with *Yersinia*, *L. pneumophila*, or *S. Typhimurium*; while caspase-4 was not necessary for IL-1β secretion (170). Thus, modulation of caspase-4 expression and activation represents a therapeutic target for systemic inflammatory response syndromes, sepsis, septic shock and related diseases (171).

### Microbial regulation of caspase-1 activation

Interestingly, intracellular *Salmonella* inhibits flagellin expression below the T-cell activation threshold to avoid triggering pyroptosis early in infection, thereby limiting inflammation and allowing bacteria to replicate intracellularly (172). Pathogens generate factors that directly suppress caspase-1 activation as well. Capase-1 has an important role in innate immune response to *P. aeruginosa*, virulent *P. aeruginosa* strains expressing ExoU can circumvent this innate immune response (173, 174). Johnston et al. discovered a poxvirus-encoded protein that inhibits caspase-1 activation by disrupting inflammasome formation (175). The IAV NS1 protein repress the cleavage of pro-IL-1β/18 and caspase-1-dependent apoptosis in primary human macrophages by controlling caspase-1 activation (176). *M. tuberculosis*-encoded Zn^2+^ metalloprotease prevents inflammasome activation and IL-1β processing (177). *Yersinia* translocates type III secretion proteins, counteracting the caspase-1 activating ability of type III secretion system, and *Yersinia* strains lacking all type III secretion proteins have an increased capacity to trigger caspase-1 (178, 179). Additionally, targeting of Rac1 by *Yersinia* effector YopE suppress caspase-1-mediated cleavage and secretion of IL-1β (180). *Actinobacillus actinomycetemcomitans* is an oral bacterium that produces a leukotoxin that selectively lyses primate neutrophils and monocytes by activating caspase-1 (181).

## Pyroptosis and diseases

### Cardivoscular diseases

Atherosclerosis is an inflammatory disease associated with endothelial dysfunction in vascular wall. The disease is characterized by the accumulation of plaques rich in fat and calcium, where cell death is triggered. Although the complete mechanism of plaque formation has not been elucidated, direct activation of the AIM2 pyroptotic pathway was found to lead to GSDMD activation and vascular smooth muscle cell pyroptosis (182). NLRP3 inflammasome activation also contributes to the vascular inflammatory response driving atherosclerosis development and progression (183). Specific inhibition of the NLRP3 inflammasome using MCC950 may be a promising therapeutic approach to inhibit the development of atherosclerotic lesions (184). MCC950 also improved cardiac function in a pig myocardial infarction model (185). IL-1β is a crucial cytokine promoting the inflammatory reaction in heart failure, Tet2 mutation in blood cells can increase atherosclerotic plaque size due to elevated IL-1β signaling in mice (186, 187). Emerging evidence suggests IL-1β as a target for atherosclerosis therapy (188, 189). In addition, enhanced cardiomyocyte (CM) NLRP3 inflammasome signaling promotes atrial fibrillation (AF) (190). The work of Takeshi et al. showed that targeting the early inflammatory response induced by CM CaMKIIδ (Ca^2+^/calmodulin-dependent protein kinase II δ) signaling could prevent progression to heart failure (191). Another study showed that aspirin exerts a protective effect against cardiovascular disease by inhibiting the activation of NLRP3 in vascular endothelial cells (192).

### Liver diseases

Substantial evidence confirms that the NLRP3 inflammasome is associated with liver failure and liver disease (193, 194). Hepatocellular carcinoma (HCC) is a primary malignancy of hepatocytes, Wei et al. showed that the expression of NLRP3 inflammasome was significantly downregulated in human HCC (195). The team further found that estrogen inhibits HCC cells through NLRP3 inflammasome-mediated pyroptosis (196, 197). Moreover, the expression of DFNA5 in HCC cells is significantly reduced, and overexpression of DFNA5 can inhibit cell proliferation (198). Hepatic steatosis or cirrhosis is closely related to the occurrence of HCC (199). Emerging evidence suggests an important role for the NLRP3 inflammasome in nonalcoholic fatty liver disease (200). As an executor of pyroptosis, GSDMD plays a key role in the pathogenesis of steatohepatitis, by controlling cytokine secretion, NF-ĸB activation, and lipogenesis (201). Interestingly, GSDMD can prevent non-infectious liver injury by regulating apoptosis and necroptosis (202).

### Lung diseases

Lung cancer is one of the leading causes of death and the most common cancer in the world (203). GSDMD was found to be upregulated in non-small cell lung cancer (NSCLC), knockdown of GSDMD attenuated tumor proliferation by promoting apoptosis and inhibiting EGFR/Akt signaling in NSCLC (204). Another study found that polyphyllin VI induced caspase-1-mediated pyroptosis by inducing the ROS/NF-κB/NLRP3/GSDMD signal axis in NSCLC (205). In addition, polydatin inhibits the proliferation and metastasis of NSCLC cells by inhibiting NLRP3 inflammasome activation through the NF-κB pathway (206). And AIM2 promotes NSCLC cell growth through an inflammasome-dependent pathway (207). LncRNA-XIST is an oncogene in various tumors and plays a regulatory role in the proliferation, invasion and metastasis of NSCLC cells (208–210). Generally, downregulation of lncRNA-XIST inhibits the development of NSCLC by activating miR-335/SOD2/ROS cascade-associated pyroptosis (211–213). Chemotherapeutic of paclitaxel and cisplatin induces pyroptosis in A549 lung cancer cells *via* activation of caspase-3/GSDME (214).

### Kidney diseases

Diabetic kidney disease (DKD) is a common clinical complication in diabetic patients and the leading cause of chronic kidney disease (CKD) in many developed and developing regions. Mitochondrial ROS promote tubular damage in diabetic nephropathy *via* the TXNIP/NLRP3/IL-1β axis (215). In turn, inhibition of NLRP3 inflammasome activation ameliorates renal injury and fibrosis in diabetic nephropathy (DN) (216), suggesting that targeting the NLRP3 inflammasome is a promising approach for the treatment of DN (217). In addition, TLR4/NF-κB signaling induces GSDMD-mediated pyroptosis in tubular cells in DKD (218), while global TLR4 knockout results in decreased renal inflammation, fibrosis and podocytopathy (219). Geniposide (GE) and Catalpol (Cat) can effectively block the oxidative stress and inflammatory response accompanying pyroptosis, thereby inhibiting the development of DN, and the mechanism may be related to the APMK/SIRT1/NF-κB pathway (220, 221). Overexpression of long non-coding RNAs MALAT1 and KCNQ1OT1 promoted high glucose (HG)-induced inflammation, oxidative stress and pyroptosis; whereas lncRNA GAS5 had the opposite effect (222–224). The findings of Pang et al. demonstrated that Irisin protects against vascular calcification (VC) by inducing autophagy and inhibiting vascular smooth muscle cell (VSMC) pyroptosis in CKD, and Irisin may serve as an effective therapeutic agent for CKD-related VC (225). Renal ischemia/reperfusion (I/R) injury is closely related to the activation of oxidative stress and pyroptosis, and the methyltransferase EZH2 may be a potential therapeutic target for renal I/R injury (226).

### Neurodegenerative diseases

Alzheimer’s disease (AD) is a neurodegenerative disorder caused by the accumulation of misfolded amyloid-β (Aβ) peptide in the brain. Only recently has neuroinflammation emerged as an important component of AD pathology (227). It is now clear that Aβ-induced activation of NLRP3 inflammasome leads to the synthesis of neurotoxic factors in microglia, which ultimately cause the development of AD (228). The connection between the NLRP3 inflammasome and the pathogenesis of AD was further confirmed using the gene-knockout approach in mice. *NLRP3*^(-/-)^ or *casp11*^(-/-)^ mice carrying AD mutations did not show loss of spatial memory (229). Clinical studies have shown that active caspase-1 is elevated in AD patients’s brains. Thus, both animal and clinical studies have demonstrated the vital role of NLRP3 inflammasome in the pathogenesis of AD, and strategies targeting the NLRP3-caspase-1 pathway are currently being explored to find treatments for AD (230–233). Parkinson’s disease (PD) is a neurodegenerative disease affecting dopaminergic motor neurons in the midbrain, and misfolding and abnormal aggregation of the neuronal protein α-synuclein are implicated in the pathogenesis of PD (234). Intracellular misfolded α-Syn can also be transferred extracellularly by exocytosis, and extracellular α-Syn aggregates induce microglial activation and astrocytes to secrete IL-1β (235). Chronic expression of IL-1β induces dopaminergic cell death in the substantia nigra (SN), accompanied by microglial activation and inflammatory cell infiltration (236). Furthermore, Gaia et al. observed that IL-1β secretion involves NLRP3 inflammasome activation (237). *NLRP3*^(-/-)^ mice are resistant to neurotoxin-induced death of dopaminergic neurons and display reduced serum levels of *IL-1β* and *IL-18* compared to the control mice (238). The above studies show that the dopamine signaling pathway and the NLRP3 inflammasome are mutually regulated, and inhibiting the inflammatory process of neurons will be helpful for the treatment of PD (239, 240).

## Conclusions and perspectives

More than 20 years have passed since pyroptosis was rst discovered, during which time, by exploring its underlying mechanisms and its role in disease development. We understand that pyroptosis is an inflammatory and programmed form of cell death that can be triggered by a variety of host or microbial factors, such as DNA damage and infection. As vital players in the pyroptosis pathway, NLRP3, caspase-1/4/5/11 and GSDMD are potential therapeutic targets. Although pyroptosis is becoming the focus of a variety of studies, many aspects remain to be explored, including its triggers, initiation mechanisms, and physiological functions. We have not determined what other elements are involved in the regulation of pyroptosis and the physiological roles of other members of gasdermin family during pyroptosis. Also, we lack authentic evidence to sustain a major role of pyroptosis in human pathology, and this crucial hurdle could be crossed when we witness the first clinical trial that leverage comprehension of pyroptotic pathway to favorably treat diseases.

## Author contributions

Conceptualization, YP and WC. Formal analysis, YP and JH. Funding acquisition, AC, MW and RJ. Investigation, YP. Methodology, YP. Project administration, AC and RJ. Supervision, RJ and ZY. Validation, RJ. Writing original draft, YP. All the authors have read and agreed to the published version of the manuscript.

## Funding

This work was supported by the National Natural Science Foundation of China (32172833/31902267), Sichuan Veterinary Medicine and Drug Innovation Group of China Agricultural Research System (CARS-SVDIP), and Sichuan Province Research Program (2022NSFSC0078).

## Conflict of interest

The authors declare that the research was conducted in the absence of any commercial or financial relationships that could be construed as a potential conflict of interest.

## Publisher’s note

All claims expressed in this article are solely those of the authors and do not necessarily represent those of their affiliated organizations, or those of the publisher, the editors and the reviewers. Any product that may be evaluated in this article, or claim that may be made by its manufacturer, is not guaranteed or endorsed by the publisher.

## Abbreviations

IL-1β, interleukin 1β; ICE, IL-1β-converting enzyme; PRRs, pattern recognition receptors; PAMPs, pathogen-associated molecular patterns; DAMPs, danger-associated molecular patterns; NLRs, nucleotide-binding oligomerization domain (NOD)-like receptors; ASC, apoptosis-associated speck-like protein; LRRs, leucine-rich repeats; PYD, pyrin domain; CARD, caspase activation and recruitment domain; GSDMD, gasdermin D; AIM2, absent in melanoma 2; LPS, lipopolysaccharide.
